# Multimodal Deep Learning and Foundation Models for Early Detection and Forecasting of Plant Diseases

**DOI:** 10.3390/plants15172564

**Published:** 2026-08-24

**Authors:** Teja Manda, Tianyu Huang, Yifan Ding, Size Dai, Liming Yang, Tingting Dai

**Affiliations:** 1 Co-Innovation Center for Sustainable Forestry in Southern China, Nanjing Forestry University, Nanjing 210037, China; teja.manda@njfu.edu.cn (T.M.); hty@njfu.edu.cn (T.H.); 18012393559@163.com (Y.D.); 2 College of Ecology and Environment, Nanjing Forestry University, Nanjing 210037, China; 3 College of Information Science and Technology & Artificial Intelligence, Nanjing Forestry University, Nanjing 210037, China; 13347707832@163.com

**Keywords:** plant disease detection, multimodal deep learning, foundation models, disease forecasting, convolutional neural networks, vision transformers

## Abstract

Plant diseases destroy 20–40% of global food production annually, posing a critical threat to food security for a projected population of 9.7 billion by 2050. Conventional diagnostic approaches relying on expert visual assessment are slow, costly, and unsuitable for modern agricultural scales. While deep convolutional neural networks demonstrated early promise, single-modality, image-centric systems consistently fail under real-world field conditions characterized by variable lighting, co-occurring infections, and cultivar diversity. This review synthesizes a decade of progress across four interconnected frontiers: the evolution of deep learning architectures for plant disease detection; the adaptation of foundation models including CLIP, SAM, and DINOv2 to agricultural contexts; the development of multimodal fusion frameworks integrating imagery, environmental, genomic, and hyperspectral data; and the transition from static disease diagnosis to descriptive comparison of reported metrics, which suggested that multimodal approaches frequently reported improved diagnostic performance relative to corresponding single-modality baselines, although direct cross-study comparison was limited by methodological heterogeneity. A systematic review following PRISMA guidelines identifies eligible comparative studies. Descriptive comparison of reported performance metrics across these studies indicated that multimodal approaches generally achieved higher accuracy and sensitivity than single-modality models, particularly for pre-symptomatic disease detection. Eight critical research gaps are identified, including the absence of a unified agricultural foundation model and limited climate-aware forecasting under non-stationary climate projections. A structured research agenda is proposed to accelerate translation from laboratory performance to globally equitable, field-deployable crop protection systems.

## 1. Introduction

The global agricultural sector faces mounting pressure to sustain food production for a projected population of 9.7 billion by 2050, while plant diseases continue to threaten crop productivity, food security, and agricultural sustainability [1]. Pathogens, including fungi, bacteria, viruses, oomycetes, and nematodes, are responsible for substantial yield losses worldwide, with the greatest burden falling on smallholder farming systems in developing regions [2,3]. Rapid and accurate disease diagnosis therefore remains a fundamental requirement for effective crop protection and sustainable agricultural production. Specifically, these developments should not be viewed as independent technological trajectories. Their significance lies in the way each addresses limitations encountered at another stage of the disease-analysis pipeline. Deep learning provides the core capacity for extracting disease-related visual representations, but its reliability is constrained by domain shift, limited annotations, and symptom ambiguity under field conditions [4]. Multimodal sensing reduces this ambiguity by incorporating spectral, environmental, structural, physiological, or molecular context, although it introduces new challenges in data alignment, representation learning, and fusion [5]. Foundation models can partly address these challenges through transferable representations, self-supervised pre-training, and parameter-efficient adaptation, but their agricultural utility depends on effective domain adaptation and their ability to encode disease-specific and cross-modal information. Disease forecasting builds on these diagnostic representations by incorporating temporal and epidemiological dynamics to move from recognition of existing symptoms toward estimation of future disease risk [6]. Thus, progress in one area directly influences the requirements and limitations of the others, creating an interdependent pathway from visual recognition to context-aware prediction and management.

Artificial intelligence (AI) has emerged as a transformative technology in plant pathology, supporting applications that range from image-based disease diagnosis to molecular genetic analysis, pathogen surveillance, disease forecasting, and precision crop management [7]. Among these, molecular genetic approaches utilize genomic, transcriptomic, proteomic, metabolomic, and other omics datasets to investigate host–pathogen interactions, disease resistance mechanisms, and biomarker discovery through computational analysis [8]. These studies involve fundamentally different biological questions, data structures, and analytical methodologies from those used in computer vision-based disease recognition. Accordingly, the present review focuses primarily on AI approaches for plant disease detection and forecasting based on computer vision, multimodal sensing, and foundation models, while molecular genetic studies are discussed only where they complement multimodal prediction frameworks or provide biologically meaningful information for disease interpretation. Historically, plant disease diagnosis has relied on expert visual assessment, a time-consuming, costly, and geographically constrained approach that is increasingly inadequate for modern agricultural systems. Early machine learning methods, including support vector machines (SVMs) and random forests, represented an important step toward automated disease diagnosis but depended heavily on handcrafted image features and showed limited robustness across diverse crops and environmental conditions [9]. The introduction of deep convolutional neural networks (CNNs), particularly architectures such as AlexNet and GoogLeNet, marked a major transition by enabling automatic feature learning directly from image data [10]. Although these approaches substantially improved disease recognition under controlled laboratory conditions, their performance frequently deteriorated in real agricultural environments characterized by variable illumination, occlusion, mixed infections, and cultivar diversity [11]. These limitations exposed the inherent constraints of single-modality image analysis and motivated the development of multimodal learning frameworks capable of integrating complementary information from imaging, environmental monitoring, remote sensing, and, where appropriate, molecular data. The emergence of transformer architectures, self-supervised learning, and large-scale foundation models has further accelerated progress in agricultural AI. Vision-language models such as CLIP, segmentation frameworks such as the Segment Anything Model (SAM), and self-supervised representation learning approaches have demonstrated the potential to improve model generalization, reduce annotation requirements, and facilitate adaptation across diverse agricultural applications [12,13]. Nevertheless, these models require careful domain-specific adaptation because general-purpose representations do not inherently capture the subtle visual, spectral, and biological characteristics associated with early-stage plant disease symptoms. Against this background, recent research has progressively evolved from isolated image classification toward integrated intelligent systems that combine multimodal sensing, transferable foundation models, and predictive analytics. Rather than treating disease diagnosis as a single classification task, these approaches increasingly incorporate environmental conditions, temporal dynamics, and complementary biological information to support early warning and decision-making throughout the disease development process.

This review synthesizes advances across four interconnected themes: (i) the evolution of deep learning methods for plant disease detection [14], (ii) the adaptation of foundation models to agricultural applications [15], (iii) multimodal learning frameworks integrating imaging, environmental, sensor, and complementary biological data [16], and (iv) AI-driven disease forecasting systems that extend diagnosis toward predictive crop health management [16]. Following a systematic review conducted according to the PRISMA 2020 guidelines (Supplementary Material), we critically evaluate the current state of the field, identify key methodological and practical challenges, and present a unified conceptual framework that links disease detection, multimodal sensing, foundation models, and forecasting into a continuous progression toward intelligent, proactive plant health management. Unlike previous reviews that examine these topics independently, this review emphasizes their interdependence and highlights how their integration is reshaping the transition from reactive disease diagnosis to predictive and decision-oriented crop protection systems.

## 2. Materials and Methods

### 2.1. Review Design

**Figure 1:**
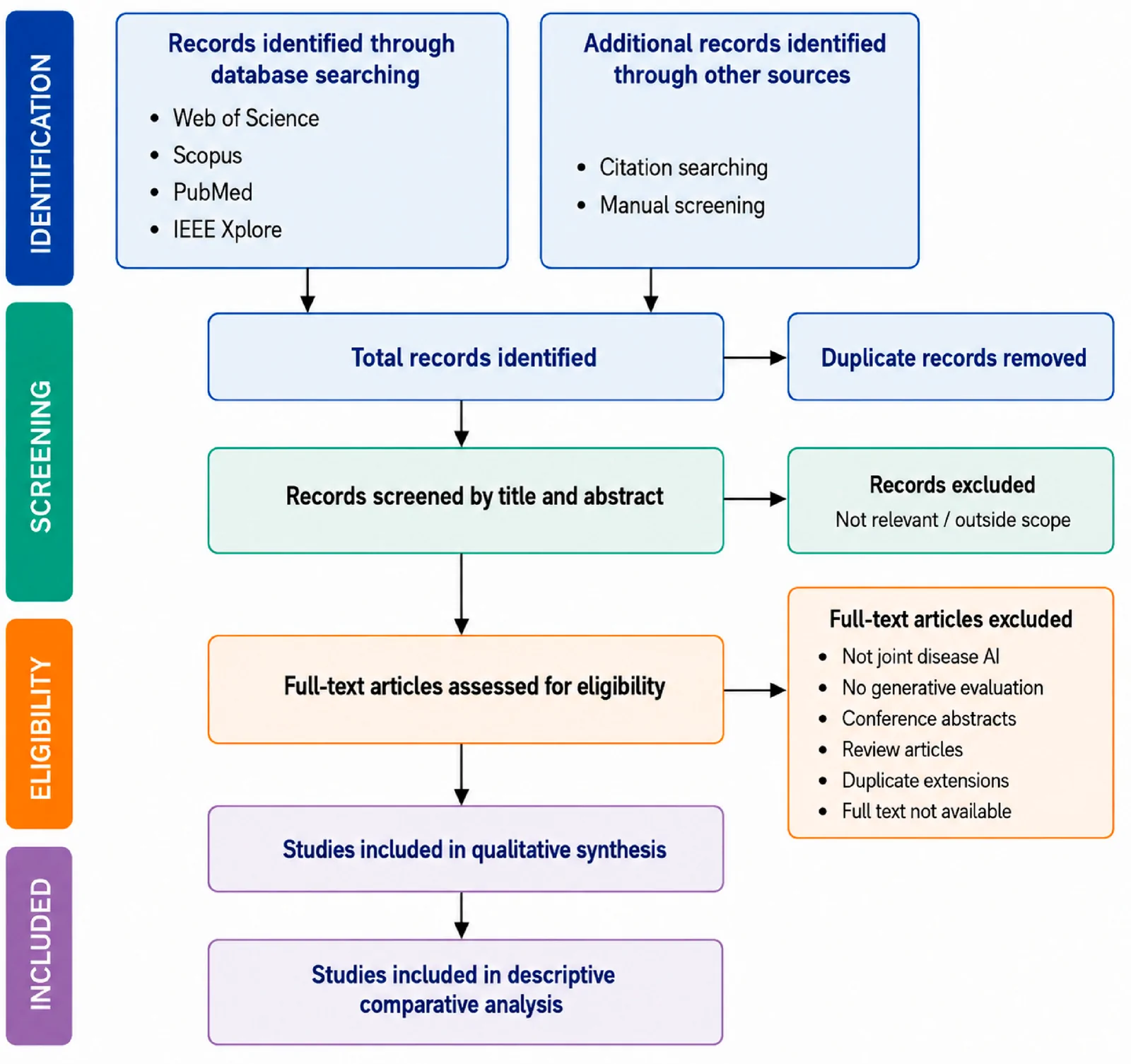
PRISMA-based flow diagram of the literature identification and selection process. Records were identified through Web of Science Core Collection, Scopus, PubMed, and IEEE Xplore Digital Library, with additional studies located through citation searching and manual screening. Following duplicate removal, records underwent title and abstract screening, full-text eligibility assessment, qualitative synthesis, and descriptive comparative analysis.

This review was conducted in accordance with the Preferred Reporting Items for Systematic Reviews and Meta-Analyses (PRISMA) 2020 guidelines, and the completed PRISMA 2020 checklist is provided in Supplementary Table S1, which identifies eligible comparative studies to ensure a transparent, reproducible, and systematic synthesis of the current literature. The objective was to comprehensively evaluate recent advances in artificial intelligence (AI) for plant disease detection and forecasting, with particular emphasis on deep learning architectures, multimodal learning, foundation models, disease forecasting systems, and edge AI applications in precision agriculture. The review process consisted of four sequential stages: literature identification, title and abstract screening, full-text eligibility assessment, and qualitative evidence synthesis. The complete study illustrates the selection and identification process in Figure 1.

### 2.2. Literature Search Strategy

A comprehensive literature search was conducted using four internationally recognized electronic databases: Web of Science Core Collection, Scopus, PubMed, and IEEE Xplore Digital Library. The search covered studies published between January 2016 and March 2026, corresponding to the period of rapid development and application of deep learning, transformer architectures, and foundation models in plant disease diagnosis and forecasting. Additional records were identified through citation searching and manual screening of the reference lists of relevant review articles and highly cited primary studies. The search strategy combined controlled vocabulary, where available, with free-text keywords using Boolean operators. Search terms included combinations of plant disease, crop disease, deep learning, machine learning, artificial intelligence, multimodal learning, foundation model, CLIP, Segment Anything Model, Vision Transformer, disease forecasting, and early detection. Database-specific syntax and indexing terms were adapted as necessary while maintaining consistent search concepts across all four databases. The identification, screening, eligibility assessment, and inclusion procedures are summarized in Figure 1.

### 2.3. Study Selection

All retrieved records were exported into EndNote 21 for reference management and automatic duplicate removal, followed by manual verification to ensure accuracy. Two independent reviewers screened the titles and abstracts of all identified records according to predefined eligibility criteria. Publications considered potentially relevant were subsequently subjected to full-text evaluation. Any disagreements regarding study eligibility were resolved through discussion until consensus was achieved. The complete screening procedure followed the PRISMA 2020 framework and is summarized in Figure 1.

### 2.4. Eligibility Criteria

The eligibility criteria for this review were defined to ensure that only studies providing substantive and methodologically comparable evidence were included. Studies were considered eligible if they were published in peer-reviewed scientific journals between 2016 and 2026, written in English, and investigated artificial intelligence applications for plant disease diagnosis, segmentation, classification, detection, or forecasting. Eligible studies evaluated machine learning, deep learning, transformer-based, multimodal, or foundation-model approaches and reported quantitative performance metrics such as accuracy, precision, recall, F1-score, Dice coefficient, Intersection over Union (IoU), mean Intersection over Union (mIoU), Area Under the Receiver Operating Characteristic Curve (AUC), or related indices. During the screening stage, records were excluded when titles or abstracts indicated that they were not relevant to plant disease analysis or fell outside the scope of AI-based disease detection. Full-text articles were subsequently assessed for eligibility, and studies were excluded if they were conference abstracts without complete manuscripts, editorials, opinion papers, patents, duplicate publications or extended versions of previously included studies, preprints, review articles, investigations lacking quantitative evaluation metrics, or studies unrelated to plant disease diagnosis or forecasting. Publications focusing exclusively on weed detection, pest monitoring, or non-agricultural computer vision applications were also excluded unless plant disease analysis constituted a primary objective. To ensure full methodological consistency, the PRISMA diagram was revised so that all exclusion categories correspond directly to these eligibility criteria, and numerical counts for identification, screening, exclusion, and full-text assessment were corrected to accurately reflect the review workflow.

### 2.5. Data Extraction and Evidence Synthesis

For each eligible study, relevant information was systematically extracted using a standardized data collection framework. The extracted variables included publication details, crop species, disease type, geographic region, dataset characteristics, sensing modality, artificial intelligence model, data fusion strategy, evaluation metrics, principal findings, methodological strengths, and reported limitations. These data formed the basis of the comparative analyses presented throughout this review and were summarized in the corresponding tables. Because the included studies differed substantially in crop species, disease types, sensing modalities, dataset composition, and evaluation metrics, the evidence was not sufficiently homogeneous to support a formal statistical meta-analysis. Accordingly, Figure 1 now distinguishes only between the qualitative synthesis and the descriptive comparative analysis, without implying any pooled statistical aggregation. In this review, descriptive comparative analysis refers to summarizing and comparing the performance metrics reported in individual studies, without computing pooled effect sizes, confidence intervals, or heterogeneity statistics. This approach allows us to highlight broad performance trends while maintaining methodological transparency and avoiding overinterpretation of heterogeneous datasets.

### 2.6. Quality Assessment

To improve the reliability of the synthesized evidence, the methodological quality of the included studies was critically evaluated using criteria adapted from previous systematic reviews of artificial intelligence applications in agriculture. Particular attention was given to dataset transparency, reproducibility of experimental procedures, external validation, reporting of performance metrics, comparison with baseline approaches, statistical rigor, availability of implementation details, and discussion of study limitations. Studies demonstrating comprehensive methodological reporting and robust experimental validation were considered to present a lower risk of bias, whereas studies with incomplete reporting or limited validation were interpreted cautiously during evidence synthesis.

## 3. From Detection to Prediction: Evolution of AI Approaches in Plant Pathology

### 3.1. Classical Machine Learning Era

**Figure 2:**
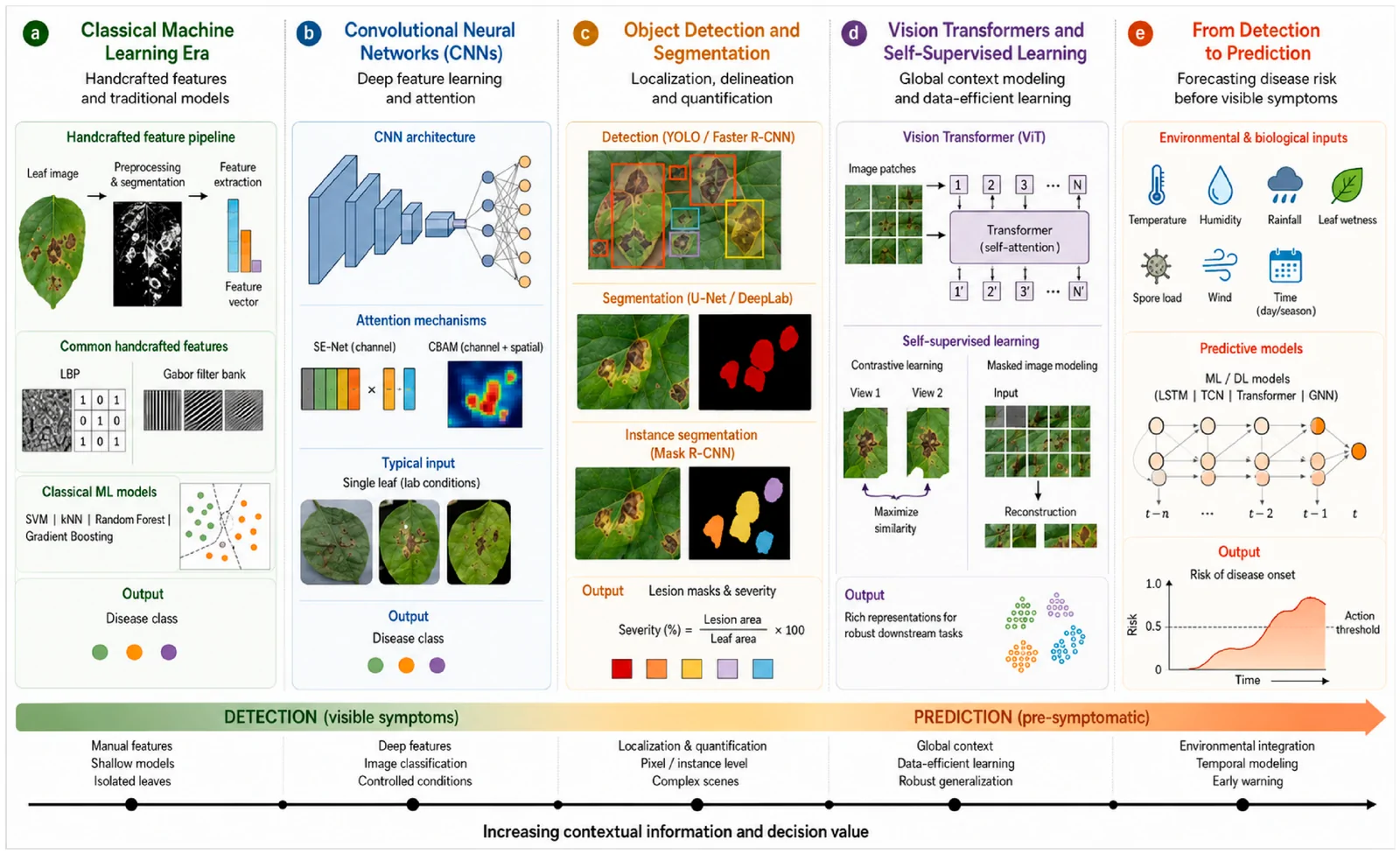
Evolution of AI approaches in plant pathology: from detection to prediction. Schematic overview of the progression from classical machine learning using handcrafted features (**a**) to CNN-based classifiers (**b**) to object detection and segmentation for localization and severity estimation (**c**) to vision transformers and self-supervised learning for robust representations (**d**) and finally to predictive modeling that forecasts disease risk before symptom appearance by integrating environmental and biological data (**e**) from detection to prediction.

Before deep learning reshaped the field, plant disease diagnosis relied on models such as support vector machines (SVMs), k-nearest neighbors (kNN), and ensemble methods, including random forests and gradient boosting approaches that required researchers to hand-engineer feature representations from raw image data (Figure 2) [17]. In the context of plant disease diagnosis, these methods operated on handcrafted visual descriptors and mathematical transformations designed by domain experts to capture specific image properties such as color distribution, surface texture [18], and geometric shape, which were extracted from pre-segmented leaf regions and supplied as fixed-length feature vectors to the classifier. Texture descriptors in particular, including Local Binary Patterns (LBPs), which encode pixel-wise relationships within local neighborhoods, and Gabor filters, which decompose image signals into frequency and orientation components analogous to mammalian visual cortex processing, were widely adopted for their ability to characterize surface irregularities associated with fungal and bacterial lesions. Understanding this era is foundational to appreciating the subsequent deep learning transition, as the brittleness of handcrafted pipelines directly motivated the shift toward learned representations.

### 3.2. Convolutional Neural Networks: The First Deep Learning Wave

CNNs changed this calculus by learning feature representations directly from image data, with early layers responding to edges and color gradients and deeper layers capturing progressively more abstract disease-relevant patterns all without the need for hand-crafted descriptors [19]. Given the chronic shortage of annotated plant disease images, transfer learning using ImageNet-pretrained weights followed by fine-tuning on agricultural datasets quickly became the default training strategy, as it enables effective adaptation of deep neural networks while reducing the requirement for large-scale labeled data and computational resources associated with training models from scratch [20]. Attention mechanisms, subsequently integrated into CNN architectures, extend this framework by allowing models to selectively weight the contribution of spatial regions or feature channels most relevant to the classification decision, with Squeeze-and-Excitation Networks (SE-Net) targeting channel-wise recalibration and the Convolutional Block Attention Module (CBAM) combining both channel and spatial attention [21].

Despite technical advances, a critical limitation persisted: CNN-based systems were designed and evaluated on isolated leaf images under controlled lighting, a setting that does not reflect the complexity of field photography, where partial occlusion, mixed-stage lesions, multiple co-occurring infections, and variable illumination are the norm rather than the exception [22]. Comparative studies demonstrated accuracy drops of 30–50 percentage points when PlantVillage-trained CNNs were applied to real field images, exposing a fundamental domain shift problem that image augmentation alone could not resolve [23].

### 3.3. Object Detection and Segmentation Advances

Object detection refers to the computer vision task of simultaneously localizing and classifying multiple objects within an image, typically producing bounding box coordinates alongside class labels and confidence scores, with modern approaches including single-stage detectors such as YOLO and two-stage detectors such as Faster R-CNN, which differ in their trade-off between inference speed and localization precision [24,25]. Semantic segmentation extends this further by assigning a class label to every individual pixel in an image, while instance segmentation as implemented in architectures such as Mask R-CNN additionally distinguishes between separate instances of the same class, enabling individual lesion delineation within a single infected leaf [26]. In plant pathology, these capabilities are critical because disease severity assessment requires not merely identifying the presence of a pathogen but quantifying the proportion of tissue affecting a metric that whole-image classification cannot provide.

### 3.4. Vision Transformers and Self-Supervised Learning

Vision Transformers (ViTs) adapted the self-attention mechanism, originally developed for natural language processing, to image recognition tasks by representing an input image as a sequence of fixed-size patches. These patches are linearly embedded and processed as sequential tokens through multi-head self-attention layers [27]. This mechanism allows the model to dynamically weight relationships between any two patches regardless of their spatial distance, capturing global contextual dependencies that are structurally inaccessible to convolutional operations bounded by local receptive fields [28]. Self-supervised learning, in parallel, refers to a class of representation learning methods in which a model is trained on pretext tasks derived from the structure of unlabeled data itself such as predicting masked regions or maximizing agreement between augmented views of the same image without requiring human-annotated labels [29].

### 3.5. The Key Transition: From Detection to Prediction

Rather than waiting for symptoms to appear, predictive models estimate when and where disease will emerge by linking environmental measurements (temperature, humidity, leaf wetness, and rainfall) to the biological timings of infection cycles, latent periods, and spore release [30,31]. Recent forecasting studies demonstrate that deep temporal models can predict disease spread several days in advance. For example, the study reported in Ref. [32] used an LSTM-based spatiotemporal architecture to forecast wheat rust risk across multiple districts in Northern India, achieving over 90% accuracy in regional risk classification.

Diagnosing disease after symptoms appear is, in an important sense, already too late. Their function is to identify visible symptoms, physiological abnormalities, or pathogen-associated signatures once the disease is established within plant tissues. Faster and more accurate diagnosis is unquestionably valuable, but it does not change the underlying reality: by the time a classifier flags an infected leaf, the pathogen has already established itself. In many crop systems, particularly those involving rapidly spreading fungal and oomycete pathogens, this delay can result in substantial yield and quality losses before corrective measures are implemented [33]. Forecasting systems attempt to overcome this limitation by estimating disease risk before symptom expression. Instead of relying solely on symptomatic evidence, these models analyze the interactions among environmental variables, host susceptibility, pathogen biology, and agronomic practices to predict the probability of future outbreaks [30]. Such predictive capability enables preventive management strategies, including optimized fungicide scheduling, irrigation regulation, resistant cultivar deployment, and targeted field monitoring. The conceptual shift resembles developments in predictive medicine, where emphasis has moved from treating established disease toward anticipating and preventing pathological progression [34].

This approach extends the established discipline of epidemiological modeling, in which mechanistic relationships between weather conditions and disease progression have long been formalized in warning systems and degree-day models, by integrating machine learning methods capable of capturing nonlinear, high-dimensional interactions that resist explicit mathematical formulation [35]. The distinction between detection and prediction is therefore not merely temporal but conceptual: whereas detection systems function as diagnostic instruments applied at the point of visible symptom expression, predictive systems operate upstream of that threshold, providing decision support during the latent phase when preventive intervention remains agronomically and economically viable. Although these developments substantially improved disease recognition, most approaches remained centered on visual diagnosis after symptom appearance. This limitation highlighted the need for more generalizable learning frameworks capable of transferring knowledge across crops, diseases, and imaging conditions. These challenges motivated the emergence of foundation models, which extend conventional deep learning by leveraging large-scale pretraining and transferable representations.

## 4. Foundation Models in Plant Disease Analysis

### 4.1. Defining Foundation Models in an Agricultural Context

**Figure 3:**
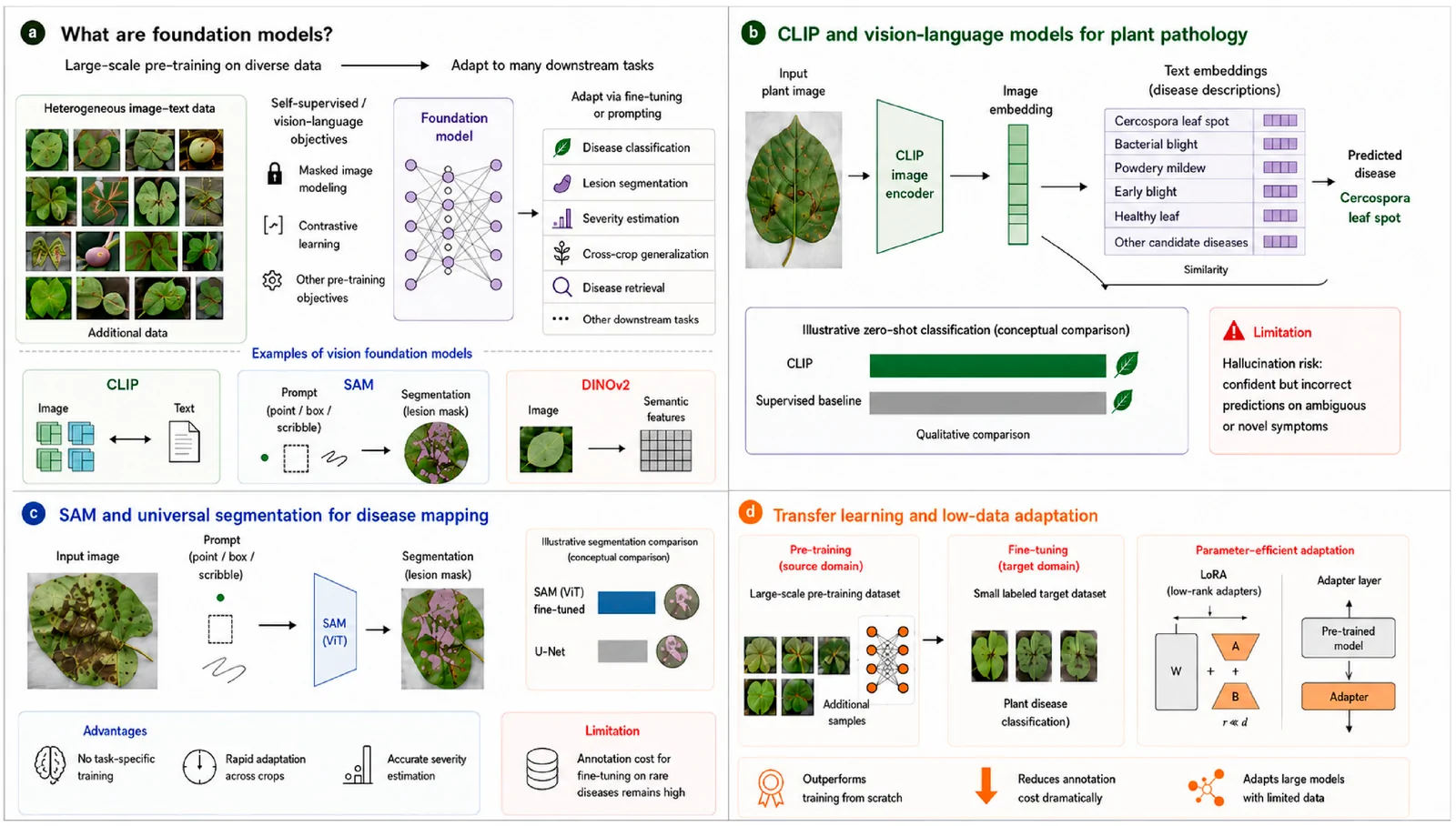
Foundation models and adaptation strategies for plant disease analysis. (**a**) Conceptual overview of foundation models, illustrating large-scale pre-training on heterogeneous image-text data and adaptation to downstream plant pathology tasks, with representative vision foundation models including CLIP, SAM, and DINOv2. (**b**) Example workflow of CLIP-based vision-language modeling for plant disease recognition, showing alignment between plant image embeddings and textual disease descriptions for zero-shot or prompt-based classification. (**c**) Schematic use of SAM for universal lesion segmentation and disease mapping from prompt-guided plant images. (**d**) Transfer learning and low-data adaptation strategies, including fine-tuning and parameter-efficient approaches, for adapting large pre-trained models to plant disease classification under limited labeled data. Comparative bars shown in panels (**b**,**c**) are conceptual and intended to illustrate qualitative differences rather than source-specific benchmark values.

Foundation models are trained on an enormous scale on diverse, unlabeled data and then adapted to specific tasks through fine-tuning or prompting, often with far less task-specific data than conventional supervised training would require (Figure 3) [36]. In the vision domain, landmark foundation models include CLIP (Contrastive Language-Image Pre-training), which aligns image and text representations; the Segment Anything Model (SAM), which enables universal object segmentation; and DINOv2, which produces high-quality semantic features through self-supervised ViT pre-training [37]. In plant pathology, these models offer a potential resolution to the persistent tension between model capacity and labeled data availability.

### 4.2. CLIP and Vision-Language Models for Plant Pathology

CLIP-based approaches enable zero-shot and few-shot disease classification by matching plant image embeddings to textual disease descriptions without requiring class-specific training examples. Recent studies have demonstrated that CLIP-based vision-language models exhibit strong zero-shot and few-shot classification capabilities in plant disease recognition. Their principal advantage lies in the ability to generalize across previously unseen crop–disease combinations through joint image-text representation learning. Nevertheless, domain-specific fine-tuning remains important to improve robustness and reduce misclassification when models are applied under diverse field conditions. This capacity for generalization to unseen disease–crop combinations is particularly valuable given the impossibility of collecting labeled training data for all crop–disease permutations encountered in global agriculture [38]. However, CLIP models exhibit hallucination risks, producing confident but incorrect classifications when presented with ambiguous or novel symptom presentations, and require careful calibration for deployment in high-stakes agricultural decision systems.

### 4.3. SAM and Universal Segmentation for Disease Mapping

The Segment Anything Model (SAM), released by Meta AI in 2023, provides interactive and automatic image segmentation without task-specific training, using prompt-based specification of the object of interest [39]. In plant pathology applications, SAM enables rapid lesion boundary delineation and severity estimation across crops and disease types without requiring disease-specific segmentation training [40]. Recent investigations have shown that the Segment Anything Model can be effectively adapted for plant disease lesion segmentation. Compared with conventional segmentation approaches, SAM-based frameworks facilitate rapid and flexible lesion delineation while substantially reducing manual annotation requirements. However, high-quality annotated datasets are still essential for reliable domain-specific adaptation [41]. The primary limitation remains annotation cost for fine-tuning: while SAM reduces the total data requirement, the production of even small high-quality segmentation datasets for rare tropical diseases remains resource-intensive.

### 4.4. Transfer Learning and Low-Data Adaptation

Transfer learning, the practice of initializing a model with weights pre-trained on a large source dataset and subsequently fine-tuning it on a smaller target dataset, has become a widely adopted training strategy in plant disease AI. ImageNet pre-training can facilitate model adaptation to agricultural classification tasks while reducing the amount of task-specific labeled data required [15]. More recently, parameter-efficient fine-tuning approaches, including LoRA (Low-Rank Adaptation) and adapter layers, have provided additional strategies for adapting large pre-trained models to specialized agricultural applications [42]. Despite their strong generalization potential, foundation models alone cannot fully characterize the complexity of plant disease development because visual symptoms represent only one component of the disease process. Disease progression is also influenced by environmental conditions, host genetics, and physiological responses. These considerations have driven increasing interest in multimodal learning frameworks capable of integrating complementary sources of information for more comprehensive disease assessment.

## 5. Multimodal Learning for Plant Disease Detection

### 5.1. The Case for Multimodality in Plant Pathology

Single-modality AI systems, particularly those relying exclusively on RGB imagery, provide an incomplete representation of plant health under field conditions. Although image-based models can achieve high performance on controlled datasets, their accuracy often declines when applied to images containing variable illumination, complex backgrounds, occlusion, or unfamiliar symptom expressions [43]. Plants may also experience several biotic and abiotic stresses simultaneously, while insect feeding, nutrient deficiencies, mechanical injury, and environmental extremes can resemble or obscure pathogen-induced lesions [44]. Consequently, image-only models may associate non-pathogenic damage with disease or fail to distinguish co-occurring stress responses, thereby reducing diagnostic specificity [45]. Consequently, image-only models may associate non-pathogenic damage with disease or fail to distinguish co-occurring stress responses, thereby reducing diagnostic specificity [46]. Hyperspectral and thermal sensing can detect pre-symptomatic alterations in pigmentation, water status, photosynthetic activity, and canopy temperature. Environmental variables, including temperature, humidity, rainfall, and leaf wetness, add epidemiological context, while textual descriptions and structured disease knowledge can provide semantic information that is not directly encoded in image pixels [42]. Integrating these complementary sourceRGB imaging remains valuable because it is accessible and captures visible color, texture, and lesion morphology. It can therefore produce a more comprehensive and context-aware assessment of crop health.

### 5.2. Data Modalities and Sensor Systems

Multimodal plant disease detection draws on several complementary data sources. RGB and near-infrared imaging provide information on visible symptoms, canopy reflectance, and vegetation indices [47]. Hyperspectral systems capture narrow spectral bands that can reveal physiological and biochemical changes associated with disease development. LiDAR and depth-sensing approaches provide three-dimensional information on canopy architecture and disease-associated structural changes, complementing two-dimensional imaging by capturing features such as wilting, deformation, and foliage loss. Environmental sensors provide temperature, humidity, rainfall, and leaf-wetness measurements associated with infection and disease progression [48]. Textual descriptions and knowledge graphs provide another modality by encoding symptom terminology, disease relationships, and expert knowledge. ITK-Net integrated disease images, textual descriptions, and a crop–disease knowledge graph to support both classification and semantic interpretation. Genomic, transcriptomic, and pathogen-genotyping data may further contribute information on host susceptibility and pathogen variation, although their direct integration with field imaging remains an emerging research direction. Across these studies, complementary modalities provide information that is difficult to obtain from RGB imagery alone. Thermal sensing captures disease-associated changes in leaf or canopy temperature, while hyperspectral and fluorescence measurements reveal alterations in reflectance, pigmentation, photosynthetic activity, and other physiological responses that may precede visible symptoms. Integration with environmental or molecular information further adds epidemiological and biological context to disease assessment. The value of multimodal sensing therefore lies not simply in increasing the number of inputs but in combining complementary signals that characterize different aspects and stages of host–pathogen interactions. Representative multimodal sensing approaches and their diagnostic contributions are summarized in Table 1. These studies illustrate how thermal, hyperspectral, fluorescence, and molecular information can complement RGB imagery by capturing physiological, biochemical, and host-related responses that are not readily represented by visible symptoms alone.

**Figure 4:**
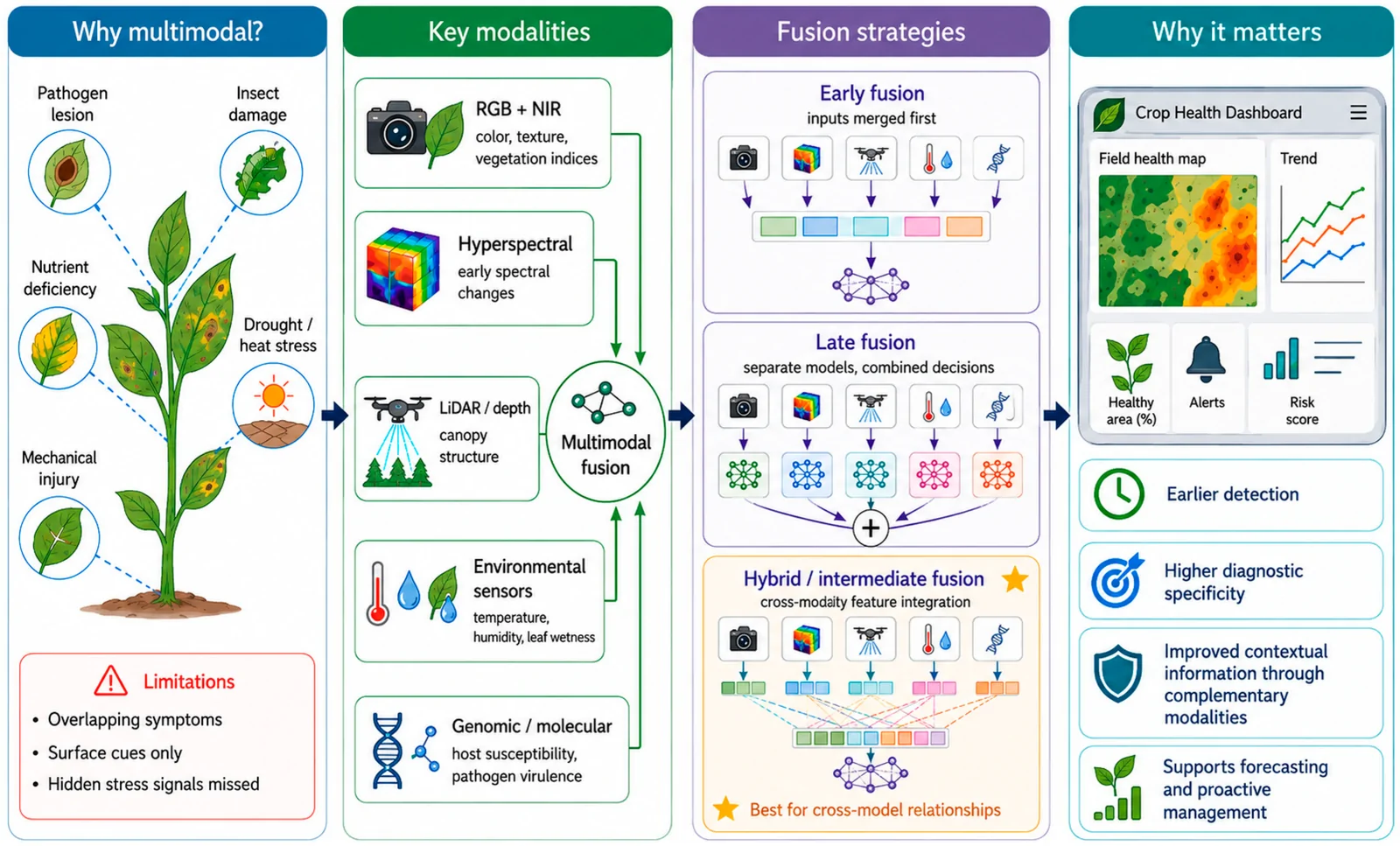
Conceptual overview of multimodal learning for plant disease detection. The framework illustrates the limitations of image-only diagnosis, the complementary contributions of visual, spectral, structural, environmental, and biological data, and their integration through early, late, and intermediate fusion strategies. Multimodal integration supports earlier and more context-aware disease assessment and provides a basis for subsequent forecasting and proactive crop-health management.

Because these data sources differ substantially in spatial resolution, temporal frequency, dimensionality, and biological meaning, their effective integration depends strongly on the fusion strategy used to combine them. The complementary roles of these data sources and their integration through early, late, and intermediate fusion strategies are conceptually summarized in Figure 4.

### 5.3. Multimodal Fusion Architectures

Multimodal fusion is generally implemented at the input, feature, or decision level. Early fusion combines aligned inputs before feature extraction and is most appropriate when the modalities have compatible spatial or temporal structures. In greenhouse tomato diagnosis, low-level fusion of visible/near-infrared and near-infrared spectral data improved the early detection of *Cladosporium fulvum* compared with several models constructed from individual spectral blocks [56].

Late fusion processes each modality independently and combines the resulting predictions through averaging, voting, or learned weighting [57]. This approach accommodates heterogeneous inputs, but it does not directly model interactions between modalities during feature extraction. Moreover, tolerance to unavailable sensors should not be assumed unless modality dropout or missing-input conditions have been included in model training and evaluation. Intermediate or hybrid fusion combines modality-specific representations before final classification, allowing the model to retain specialized features while learning cross-modal relationships. Multi-ResNet34 used feature-level fusion to integrate tomato images with environmental parameters, while ITK-Net jointly represented disease images, textual descriptions, and knowledge-graph information for tomato and cucumber disease identification. RustQNet similarly combined RGB imagery, multispectral information, and expert-informed vegetation indices for quantitative estimation of wheat stripe rust severity [58]. However, limited paired data can also hinder effective cross-modal alignment, particularly when linking textual symptom descriptions with corresponding visual disease features. Thus, multimodal learning may reduce the effects of small datasets while simultaneously increasing the need for well-aligned, representative paired samples.

### 5.4. Advantages over Single-Modal Systems

The progression from conventional image-based architectures to multimodal and foundation-model approaches is summarized in Table 2, highlighting their principal data requirements, disease applications, and methodological limitations. Representative studies indicate that multimodal integration can improve disease classification, early diagnosis, severity estimation, and contextual interpretation compared with corresponding single-source approaches [56,59]. The principal advantage is complementarity: spectral information can reveal pre-visual physiological changes, environmental data can provide epidemiological context, structural sensors can characterize canopy deformation, and textual or knowledge-based inputs can support interpretable disease reasoning. Nevertheless, these findings should not be interpreted as evidence of a universal performance advantage. The published studies differ substantially in crop species, disease classes, sensor configurations, datasets, fusion strategies, and evaluation procedures. Most report task-specific comparisons rather than cross-site validation, and relatively few directly evaluate missing modalities, sensor failure, seasonal transfer, or deployment under independent field conditions. Multimodal systems should therefore be described as a promising route toward more comprehensive and context-aware disease assessment, while their operational reliability requires further multi-location, multi-season, and external validation.

## 6. Disease Forecasting and AI-Driven Decision Support

### 6.1. From Reactive Diagnosis to Proactive Risk Prediction

**Figure 5:**
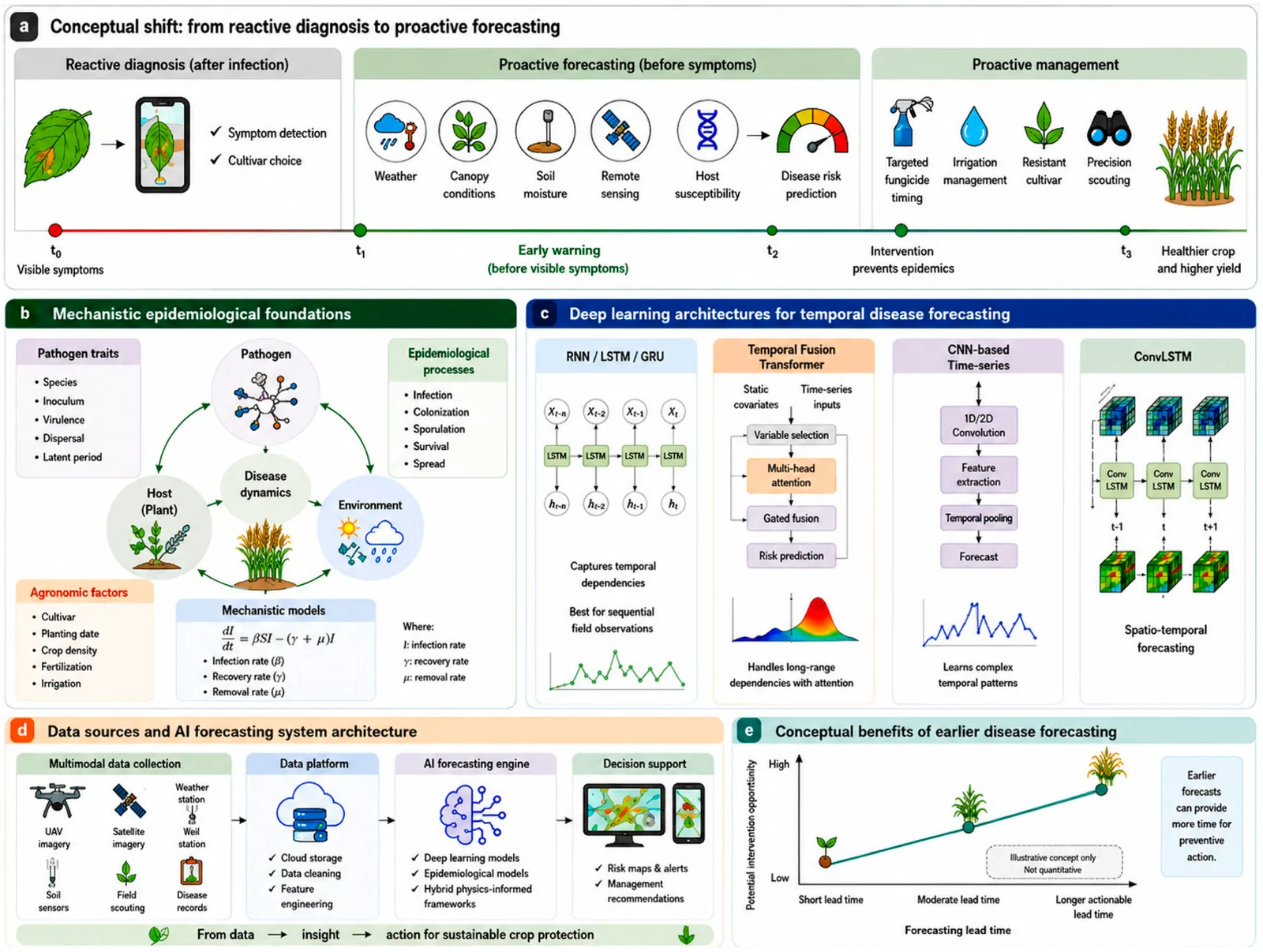
AI-driven disease forecasting and decision support for proactive crop protection: (**a**) Conceptual shift from reactive diagnosis to proactive risk prediction and management. (**b**) Mechanistic epidemiology foundations integrating pathogens, hosts, environments, and agronomic factors. (**c**) Deep learning architectures for temporal disease forecasting. (**d**) Data sources and AI forecasting system architecture. (**e**) Impact of forecasting lead time on yield protection.

Climate change is reshaping the epidemiology of many plant diseases by altering the environmental conditions that govern pathogen development, host susceptibility, and infection dynamics. Rising temperatures, elevated atmospheric CO_2_, shifting rainfall patterns, and more frequent extreme weather events are modifying the timing, intensity, and geographic distribution of numerous fungal and oomycete pathogens [68]. These changes complicate disease management by increasing uncertainty in outbreak behavior and reducing the reliability of historical disease calendars. Experimental studies have shown that elevated temperature and CO_2_ can alter pathogen infection efficiency, sporulation rates, and host susceptibility, with several fungal pathogens exhibiting accelerated epidemic development under warming scenarios. Such physiological shifts influence not only the speed of epidemic onset but also the visual progression of symptoms in the field. As illustrated in Figure 5, environmental stressors such as heat and elevated CO_2_ can modify lesion morphology, chlorotic boundary formation, and sporulation density, thereby altering the visual signatures that diagnostic systems rely upon. These climate-driven changes complicate image-based detection pipelines, which often assume stable symptom appearance across seasons and growing regions. Because climate change affects both the biological processes underlying infection and the visual expression of disease symptoms, forecasting systems must increasingly incorporate climate-aware variables. Modern predictive frameworks therefore integrate temperature, humidity, rainfall, leaf wetness, remote-sensing observations, and other environmental variables to characterize conditions associated with disease development and support more timely risk assessment.

### 6.2. Mechanistic Epidemiological Foundations

Despite rapid progress in artificial intelligence, effective disease forecasting still depends heavily on established principles of plant epidemiology. Decades of field epidemiology have established that disease outbreaks are not random; rather, they follow patterns determined by the interactions among pathogen biology, host susceptibility, and environmental conditions. These relationships provide an important mechanistic basis for disease forecasting. For many economically important diseases, the environmental conditions favoring infection, sporulation, dispersal, and disease progression have been experimentally characterized over several decades. These mechanistic insights remain essential because they provide biologically interpretable constraints that improve model reliability and reduce spurious predictions generated purely from statistical correlations [69]. Several classic pathosystems illustrate the importance of mechanistic epidemiological knowledge. Phytophthora infestans, the causal agent of potato late blight, typically requires extended leaf wetness periods and moderate temperatures for successful infection [6]. *Puccinia striiformis*, responsible for wheat yellow rust, develops most effectively under cool and humid conditions accompanied by dew formation, whereas *Magnaporthe oryzae*, the rice blast pathogen, exhibits enhanced sporulation and infection under warm nighttime temperatures combined with elevated humidity [70]. Similar environmental thresholds have been reported for grape powdery mildew, soybean rust, citrus canker, and Fusarium head blight. These experimentally validated relationships formed the basis of early rule-based warning systems such as Blitecast, NegFry, and EPIPRE, which represented some of the earliest operational disease forecasting platforms in agriculture.

Contemporary AI systems increasingly incorporate these epidemiological principles rather than relying exclusively on black-box learning. Hybrid modeling approaches combine mechanistic disease equations with machine learning architectures, allowing biological knowledge to guide predictive inference [71]. Physics-informed neural networks (PINNs), for example, embed epidemiological constraints directly into deep learning optimization frameworks, enabling models to respect known infection dynamics while still adapting to complex environmental datasets [72]. Between 2022 and 2025, several studies demonstrated that PINN-based forecasting systems improved prediction stability under limited or noisy datasets compared with purely data-driven neural networks. Another important development involves coupling epidemiological models with high-resolution environmental observations derived from remote sensing and IoT systems. Instead of using generalized regional climate averages, modern forecasting platforms increasingly rely on canopy-level microclimate measurements, including leaf wetness duration, vapor pressure deficit, and localized temperature gradients. This refinement is particularly valuable for diseases with strong microenvironmental dependence, such as downy mildews and Botrytis infections. In addition, mechanistic epidemiology has become increasingly relevant under climate change research, where predictive systems must account for shifting pathogen geographic ranges, altered overwintering behavior, and modified host–pathogen interactions under elevated temperature and CO_2_ conditions [37]. The integration of biological epidemiology with artificial intelligence therefore represents a critical direction for next-generation disease forecasting. Rather than replacing traditional plant pathology knowledge, AI systems are increasingly functioning as computational extensions of established epidemiological theory, enabling more scalable, adaptive, and spatially resolved prediction frameworks for modern agricultural systems.

### 6.3. Deep Learning Architectures for Temporal Disease Prediction

Recurrent neural networks (RNNs) and their gated variants, including LSTM and GRU, were among the earliest deep learning frameworks adopted for temporal disease forecasting. These models replaced conventional threshold-based warning systems with data-driven approaches trained on multi-year meteorological and outbreak datasets [73]. AI-based disease forecasting studies, their input data types, model architectures, and prediction outcomes are shown in Table 3. Subsequent studies between 2020 and 2024 further demonstrated the applicability of temporal deep learning across multiple cropping systems [35]. For example, an LSTM-based forecasting system for potato late blight was developed in Ref. [74] using temperature, humidity, and rainfall data, with reported prediction accuracies exceeding 90% under field conditions in India. Similarly, a GRU-based framework integrating IoT-enabled weather sensors was reported in Ref. [75] for rice blast prediction in eastern China. This approach provided earlier disease-risk warnings than conventional regression-based epidemiological models, highlighting the potential of recurrent neural networks for capturing temporal dependencies in environmental variables and supporting timely disease management. Transformer-based temporal architectures later improved forecasting performance by capturing long-range dependencies within climate time-series datasets through self-attention mechanisms [76]. In 2023, a Temporal Fusion Transformer (TFT) model was developed for wheat stripe rust forecasting using meteorological observations and remote sensing indices, achieving superior prediction stability during irregular rainfall events compared with LSTM-based approaches [32]. More recently, transformer frameworks coupled with satellite-derived vegetation indices have enabled early warning systems at regional scales. Convolutional LSTM (ConvLSTM) architectures integrate spatial convolution with temporal recurrence, allowing simultaneous modeling of disease dynamics across both space and time.

## 7. Edge AI for Field Deployment

### 7.1. The Case for On-Device Inference

The agronomic utility of AI disease systems depends critically on their accessibility to the end user the farmer operating in a field environment often characterized by limited internet connectivity, intermittent power supply, and absence of technical support infrastructure [80]. Cloud-based inference is simply not an option across much of sub-Saharan Africa and rural Asia, where network connectivity is unreliable and a diagnostic response delayed by seconds is operationally useless [81]. Running inference directly on the device whether a smartphone, a UAV, or an IoT sensor node eliminates the need for a network connection and makes real-time diagnosis genuinely feasible at the field edge. For plant disease AI, edge deployment encompasses smartphone-based diagnosis applications, UAV-embedded processing for aerial disease mapping, and IoT node-level inference on sensor platforms deployed throughout the field.

### 7.2. Model Compression for Agricultural Edge Devices

Deploying state-of-the-art deep learning models on edge hardware requires systematic compression to reduce parameter counts, memory footprint, and computational load while preserving diagnostic accuracy. The primary compression techniques applicable to agricultural AI include: (1) knowledge distillation, in which a lightweight student network is trained to replicate the output distributions of a large teacher model [82]; (2) pruning, which removes redundant weights below a magnitude threshold, typically achieving 80–90% parameter reduction with less than 2% accuracy loss for classification tasks [83]; (3) quantization, which reduces the numerical precision of weights and activations from 32-bit floating point to 8-bit integer or lower, enabling 4× memory reduction and 2–3× inference speedup on integer-optimized hardware; and (4) Neural Architecture Search (NAS), which automatically designs architectures optimized for target hardware constraints [84]. MobileNet, EfficientNet-Lite, and SqueezeNet architectures have been validated for on-device plant disease classification on Android and iOS devices, achieving 92–95% accuracy on benchmark datasets with inference times of 30–80 milliseconds [85]. Dedicated AI accelerator chips, including Google Edge TPU, Apple Neural Engine, and Qualcomm Hexagon DSP, can further accelerate inference by approximately 5–20× relative to CPU execution, enabling real-time video-rate disease scanning during field operations [86].

### 7.3. UAV-Based Precision Disease Mapping

Unmanned aerial vehicles (UAVs) equipped with RGB, multispectral, or other imaging sensors provide an effective platform for monitoring crop health over large agricultural areas and generating spatial information that can support targeted disease-management interventions [87]. The integration of edge-computing hardware with UAV platforms can further enable image processing and disease-related inference closer to the point of data acquisition, reducing dependence on continuous cloud connectivity and post-flight processing [88]. Nevertheless, operational deployment requires careful consideration of computational requirements, sensor performance, flight conditions, data-transfer constraints, and validation under representative field environments [89].

## 8. Key Challenges, Research Gaps, and Future Directions

### 8.1. Identification of Critical Research Gaps

Despite rapid advances in AI-enabled plant disease detection and forecasting, several persistent limitations continue to restrict reliable deployment under real agricultural conditions. Eight major research gaps were identified across the reviewed literature: the absence of unified agricultural foundation models, limited climate-aware disease forecasting, insufficient model interpretability, data scarcity and annotation bias, poor field generalization, computational constraints for edge deployment, weak integration with precision-agriculture systems, and the lack of standardized benchmarking and large-scale validation. To provide a consistent analytical treatment, each gap is examined according to four components: (i) its current manifestation, (ii) the underlying scientific or technical barrier, (iii) the agronomic consequence if unresolved, and (iv) a proposed research direction. This structure enables direct comparison among the gaps while linking methodological limitations to their practical implications for plant disease management.

### 8.2. Research Gap 1: Absence of Unified Agricultural Foundation Models

Most foundation models currently applied to plant disease analysis originate from general computer vision or vision-language domains and are subsequently adapted to agricultural datasets. Although models such as CLIP, SAM, and DINOv2 provide transferable representations and can reduce dependence on task-specific model development, they are not inherently trained to represent the biological, phenotypic, environmental, and disease-related variability encountered in agricultural systems. Their effective application therefore frequently requires additional fine-tuning using crop- and disease-specific datasets. Developing a unified agricultural foundation model remains challenging because existing datasets are fragmented across crop species, pathogens, geographic regions, sensing platforms, and modalities and vary considerably in annotation quality, image resolution, disease severity, and acquisition conditions. This fragmentation limits cross-crop and cross-region transferability and may prevent general-purpose models from distinguishing subtle disease symptoms from visually similar abiotic stresses. Future research should therefore prioritize agricultural foundation models pre-trained on large, diverse, and carefully curated datasets encompassing multiple crops, pathogens, growth stages, agroecological regions, and sensing modalities. Multimodal and self-supervised pre-training, combined with parameter-efficient adaptation, could provide a scalable route toward models capable of transferring agricultural knowledge across disease-detection tasks while reducing dependence on extensive task-specific annotation.

### 8.3. Research Gap 2: Lack of Climate-Aware Disease Forecasting

Current AI-based disease forecasting systems rely predominantly on historical weather observations and disease occurrence records, with variables such as temperature, relative humidity, rainfall, and leaf wetness commonly used to estimate infection risk. Although these approaches are useful for short-term prediction, models trained primarily on historical relationships may have limited capacity to anticipate disease behavior under climatic conditions outside the range represented during training. This is particularly important because climate change can modify pathogen development rates, host susceptibility, infection periods, vector activity, overwintering potential, and the geographic suitability of crop diseases [90]. Failure to account for these changes may reduce the reliability of established warning thresholds and disease calendars, particularly in regions experiencing rapid shifts in temperature and precipitation regimes. Future forecasting frameworks should therefore integrate climate projections with biological and epidemiological information. CMIP6 ensemble projections could be incorporated into AI-based models to evaluate disease risk under alternative temperature, precipitation, humidity, and extreme-weather scenarios. Combining these projections with crop phenology, host susceptibility, pathogen thermal-response functions, and mechanistic epidemiological constraints may enable scenario-dependent assessments of future disease risk. Physics-informed and hybrid mechanistic-AI models could further improve extrapolation beyond historically observed conditions by constraining predictions according to established host–pathogen environment relationships.

### 8.4. Research Gap 3: Limited Model Interpretability and Explainability

Despite improvements in predictive performance, many deep learning, multimodal, and foundation-model approaches remain difficult to interpret biologically. Attention maps and feature-attribution methods can indicate image regions associated with model predictions, but these visualizations do not necessarily establish that the model has learned biologically meaningful disease characteristics [91]. The challenge becomes greater in multimodal architectures, where a prediction may simultaneously depend on visual symptoms, spectral signatures, environmental variables, textual descriptions, and other information sources. Without understanding the relative contribution of these inputs, apparently accurate predictions may result from background artifacts, acquisition conditions, or other spurious correlations rather than pathogen-specific features. This creates a significant barrier to reliable decision support, particularly for visually similar diseases, mixed infections, and overlapping biotic and abiotic stresses. Future studies should therefore move beyond conventional saliency visualization toward biologically grounded explainability frameworks that quantify modality-specific contributions, identify influential environmental and physiological variables, and incorporate uncertainty estimates. Model explanations should also be evaluated against established plant pathological knowledge and expert assessment to determine whether the reasoning underlying a prediction is agronomically credible.

### 8.5. Research Gap 4: Data Scarcity, Annotation Challenges, and Dataset Bias

The availability and quality of training data remain major constraints on the development of reliable plant disease AI systems. Existing datasets are highly uneven in their representation of crop species, disease classes, infection stages, cultivars, and field environments. Common diseases are often represented by large numbers of images, whereas rare diseases, early infections, mixed symptoms, and geographically restricted pathogens remain poorly represented. Expert annotation is also labor-intensive and can be difficult when symptoms overlap with nutrient deficiencies, pest damage, or environmental stress. As summarized in Table 4, widely used plant disease datasets range from highly controlled laboratory image collections to field-acquired and multimodal datasets, yet each presents limitations that may restrict model generalization and real-world applicability. Multimodal learning intensifies this challenge because image, environmental, spectral, textual, or molecular observations must often be accurately paired for effective cross-modal alignment. Although complementary modalities can partly compensate for small image datasets by increasing the information available for each sample, insufficient paired observations may prevent reliable learning of relationships among modalities. Future work should therefore combine self-supervised learning, transfer learning, few-shot learning, active learning, and carefully validated synthetic-data approaches to reduce dependence on extensive manual annotation. Dataset development should simultaneously prioritize rare diseases, early infection stages, mixed stresses, geographic diversity, and transparent annotation protocols so that improvements in model performance are not achieved at the expense of representation quality.

### 8.6. Research Gap 5: Limited Field Generalization Across Crops and Agroecological Regions

A persistent limitation of plant disease AI is the discrepancy between performance reported on internally partitioned datasets and performance achieved under independent field conditions. Many studies divide images randomly into training and testing subsets collected using similar sensors, backgrounds, cultivars, locations, and environmental conditions. Such evaluation can underestimate the effects of domain shift and produce optimistic estimates of deployment performance. In practice, illumination, camera characteristics, crop variety, disease severity, canopy structure, soil background, management practices, climate, and pathogen populations can vary substantially between regions and seasons [33]. Models may consequently learn dataset-specific correlations rather than generalizable biological characteristics of disease, leading to reduced reliability when transferred to new production environments. This limitation has direct agronomic consequences because inaccurate predictions can result in missed infections or unnecessary disease-control interventions. Future studies should therefore prioritize independent external validation, including cross-location, cross-season, cross-cultivar, and cross-sensor evaluation. Domain adaptation, domain generalization, self-supervised pre-training, and invariant representation learning may further improve transferability, but their effectiveness should be assessed using geographically and temporally independent datasets rather than only random within-dataset partitions.

### 8.7. Research Gap 6: Computational and Operational Constraints for Edge AI Deployment

The increasing complexity of transformers, multimodal architectures, and foundation models has created an important tension between predictive capability and practical deployability. Large models may require substantial memory, computational resources, and energy, while multimodal systems add further processing requirements because several sensor streams must be acquired, encoded, synchronized, and fused [97]. These requirements can exceed the capabilities of smartphones, UAVs, embedded processors, and other devices commonly available in agricultural environments. Dependence on cloud computing is also problematic where network connectivity is intermittent or unavailable, particularly in remote and resource-constrained farming regions. Computational efficiency should therefore be considered during model development rather than treated solely as a post-training optimization problem. Knowledge distillation, structured pruning, quantization, lightweight architectures, neural architecture search, and parameter-efficient adaptation provide promising strategies for reducing computational requirements. However, future studies should evaluate these approaches directly on representative agricultural edge devices and report latency, memory use, energy consumption, and diagnostic reliability alongside conventional performance metrics. Adaptive multimodal systems capable of selecting only the most informative sensor inputs may provide an additional means of reducing sensing and computational costs.

### 8.8. Research Gap 7: Weak Integration Between AI Disease Prediction and Precision Farming Systems

Many AI-based plant disease studies terminate at classification, segmentation, severity estimation, or risk prediction without demonstrating how these outputs can be translated into actionable crop-management decisions. Consequently, highly accurate detection models may remain isolated analytical tools rather than functioning components of precision agriculture systems [98]. Converting a disease prediction into an appropriate intervention requires additional information concerning crop developmental stage, disease severity, spatial distribution, weather forecasts, treatment thresholds, pesticide regulations, economic considerations, and farm-management practices. These data frequently originate from different platforms and operate at different spatial and temporal resolutions, creating substantial interoperability challenges. Without integration into decision-support workflows, improvements in diagnostic accuracy may have limited impact on disease suppression or resource-use efficiency. Future systems should therefore connect disease detection and forecasting with geospatial information systems, UAV surveillance, IoT sensor networks, variable-rate application technologies, and farm-management platforms. Evaluation should also extend beyond classification metrics to determine whether AI-supported decisions improve intervention timing, reduce unnecessary pesticide use, lower economic losses, and provide measurable agronomic benefits while retaining appropriate expert oversight.

### 8.9. Research Gap 8: Lack of Standardized Benchmarking and Large-Scale Validation Frameworks

Comparative assessment of plant disease AI remains difficult because published studies differ substantially in datasets, crop species, disease classes, preprocessing procedures, train-test partitioning, evaluation metrics, and experimental conditions. As a result, performance values reported by different studies cannot always be interpreted as direct evidence that one architecture or sensing strategy is superior to another. The absence of standardized, representative benchmarks is particularly problematic because many datasets contain controlled or semi-controlled images and do not adequately capture the diversity encountered during field deployment. External testing and prospective multi-season validation also remain comparatively limited [99]. These inconsistencies make it difficult to distinguish genuine methodological advances from improvements arising from favorable datasets or evaluation procedures and restrict reproducibility across research groups. Future efforts should therefore establish open, multi-institutional benchmarking resources that include diverse crops, diseases, cultivars, severity stages, sensing systems, agroecological regions, and independent field observations. Standardized evaluation should extend beyond overall accuracy to include class-specific performance, uncertainty and calibration, computational efficiency, resistance to domain shift, and external generalization. Multi-location and multi-season validation will be particularly important for determining whether AI systems remain reliable when transferred from experimental datasets to changing agricultural environments. Collectively, these gaps demonstrate that advancing AI for plant disease management requires more than incremental improvements in classification performance. Domain-specific foundation models, multimodal integration, climate-aware prediction, biological interpretability, representative datasets, cross-domain generalization, computational efficiency, precision-agriculture integration, and standardized validation are closely connected challenges rather than isolated research problems. Progress toward reliable plant-health intelligence will therefore depend on addressing these limitations jointly, enabling the transition from experimental disease-recognition systems toward scalable platforms capable of supporting early diagnosis, disease forecasting, and evidence-based crop protection across diverse agricultural environments.

## 9. Conclusions

This review highlights the extraordinary evolution of AI-driven plant disease management from early CNN-based classification models to multimodal foundation models and demonstrates a fundamental shift from reactive disease diagnosis to proactive, climate-aware prediction and intelligent decision support. Recent advances in vision transformers, foundation models, multimodal data fusion, and AI-driven forecasting have substantially improved disease detection accuracy, early warning capabilities, and rigor under real-world agricultural conditions while addressing longstanding challenges such as limited labeled datasets and heterogeneous field environments. Nevertheless, significant barriers remain before these technologies can achieve widespread practical adoption. Key challenges include the absence of unified agricultural foundation models, limited cross-domain generalization across crops and environments, underrepresentation of tropical and subsistence crops, fragmented multimodal datasets, insufficient model interpretability, and weak integration with precision agriculture systems. Overcoming these limitations will require coordinated investments in open and standardized datasets, internationally accepted evaluation frameworks, interdisciplinary collaboration, and participatory development involving farmers, plant pathologists, AI researchers, and policymakers. Looking forward, the future of plant disease management lies in the convergence of multimodal sensing, explainable AI, climate-informed disease forecasting, and autonomous decision-support systems capable of continuously learning from diverse field observations and delivering timely, adaptive management recommendations. Such integrated AI ecosystems have the potential to reduce crop losses, optimize agrochemical inputs, improve resource-use efficiency, and enhance agricultural adaptability under increasingly variable climatic conditions. Ultimately, the long-term impact of AI in plant pathology will be determined not only by continued improvements in predictive accuracy but also by the development of scalable, equitable, and field-ready solutions that can be effectively deployed across diverse cropping systems, contributing to sustainable agriculture and global food security. Future AI systems should move beyond recognizing isolated disease symptoms toward comprehensive plant health assessment capable of distinguishing pathogen infection from insect damage, abiotic stress, and other co-occurring factors commonly encountered under field conditions.

## Figures and Tables

**Table 1:** Representative multimodal sensing approaches and their diagnostic contributions.

Modalities Used	Crop/Disease Context	Advantage over Single-Modal	Study (Year)
RGB + thermal IR	Wheat leaf rust, grapevine downy mildew	Captured disease-associated physiological and thermal responses before clearly visible symptom development, including alterations in stomatal activity and leaf or canopy temperature	[49]
RGB + hyperspectral	Tomato bacterial spot, maize fungal infection	Improved early-stage disease discrimination by combining visible symptoms with spectral changes associated with pathogen-induced physiological and biochemical responses	[50,51]
RGB + Fluorescence	Soybean sudden death syndrome	Increased sensitivity to infection-associated changes in chlorophyll fluorescence and photosynthetic function, supporting earlier recognition of disease-related stress	[52,53]
RGB + Genomic/Transcriptomic	Rice blast	Combined phenotypic information with molecular indicators associated with host susceptibility and resistance, providing complementary information for disease prediction	[54,55]

**Table 2:** Representative AI models for plant disease detection and their data requirements, applications, and methodological limitations.

Model Type	Crop/Disease	Data Type	Limitation	Study/Year Reference
CNN (AlexNet/GoogLeNet)	Tomato leaf disease	RGB images	Lab conditions only; poor field generalization	[60]
ResNet-50 + attention	Rice/blast, sheath blight	RGB images	Single crop; static diagnosis	[61]
Vision Transformer (ViT)	Wheat/yellow rust	RGB + NIR	Small dataset; limited generalization	[62]
EfficientNet-B4	Tomato/multiple diseases	RGB images	No temporal or weather data	[63]
Multimodal CNN + LSTM	Maize/leaf blight	Image + weather	High compute cost	[64]
ViT + hyperspectral fusion	Soybean/soybean mosaic virus	RGB + spectral	Expensive sensors; dataset bias	[65]
CLIP-based foundation model	Multiple crops/multiple	Image + text	Hallucination risk; needs fine-tuning	[66]
SAM + ViT (transfer learning)	Cassava/mosaic virus	RGB images	Annotation cost; domain shift	[40]
Multimodal Transformer	Multi-crop/disease risk	Image + genomic + env	Data fusion complexity	[67]

**Table 3:** Representative AI-based plant disease forecasting approaches, applications, contributions, and methodological limitations.

Model Type	Crop/Disease	Data Type	Limitation	Reference
LSTM-based forecasting model	Wheat/rust	Weather + temporal infection cycles	Requires dense time-series data	[77]
GRU-based forecasting model	Litchi/downy mildew	Humidity + leaf wetness + temperature	Limited generalization across regions	[78]
Multimodal Transformer (forecasting)	Multi-crop/disease risk	Image + environmental + phenology + temporal data	Complex fusion; high compute	[67]
Hybrid epidemiological–ML model	Potato/late blight	Weather + spore release + ML risk scoring	Requires pathogen-specific parameters	[79]

**Table 4:** Representative datasets used in AI-based plant disease analysis and their principal limitations for model development and field generalization.

Dataset Name	Data Type	Conditions	Key Limitation	Reference
PlantVillage	RGB leaf images	Lab (controlled)	No field noise; clean backgrounds; no temporal variation	[92]
Plant Pathology 2020	RGB images	Field/semi-field	Single crop; moderate class imbalance	[93]
Edge RiceLeafDis	RGB images	Lab	Small sample size; no environmental context	[94]
FLAIR (Remote Sensing)	Multispectral satellite	Field	Coarse spatial resolution; no disease-level labels	[95]
WheatNet	RGB + meteorological	Field	Small temporal window; region-specific bias	[96]

## Data Availability

No new data were created or analyzed in this study. Data sharing is not applicable to this article.

## Supplementary Materials

The following supporting information can be downloaded at: https://www.mdpi.com/article/10.3390/plants15172564/s1.

## Abbreviations

The following abbreviations are used in this manuscript:

ML	Machine Learning
AI	Artificial Intelligence
CNN	Convolutional Neural Network
ViT	Vision Transformer
RNN	Recurrent Neural Network
LSTM	Long Short-Term Memory
GRU	Gated Recurrent Unit
TFT	Temporal Fusion Transformer
ConvLSTM	Convolutional Long Short-Term Memory
NAS	Neural Architecture Search
PINN	Physics-Informed Neural Network
