# Supplementary material for: Multimodal Deep Learning and Foundation Models for Early Detection and Forecasting of Plant Diseases
Source: Plants (Basel). 2026 Aug 24;15(17):2564. doi: 10.3390/plants15172564 (PMC13567198; doi:10.3390/plants15172564)
Supplement: Supplementary file 1 [file plants-15-02564-s001.zip › plants-4451944-supplementary.pdf]

PRISMA 2020 Checklist

Table S1

Section / Topic	Item	Checklist	Location where item is reported
**TITLE**			
Title	1	Identify the report as a systematic review.	Title, The article is identified as a Review; the systematic-review design is stated in the Abstract and Section 2.1
**ABSTRACT**			
Abstract	2	Provide an abstract that reports the key elements recommended by PRISMA 2020 for abstracts.	Abstract, p. 2.
**INTRODUCTION**			
Rationale	3	Explain the review in the context of existing knowledge.	Section 1, Introduction
Objective	4	State the review objective(s) or question(s) explicitly.	Section 1, final paragraph
**METHODS**			
Eligibility criteria	5	Specify inclusion and exclusion criteria and how studies were grouped for synthesis.	Section 2.4, Eligibility Criteria; synthesis approach in Section 2.5
Information source	6	List all information sources searched and report the date each source was last searched.	Section 2.2, Literature Search Strategy. Web of Science, Scopus, PubMed, IEEE Xplore, citation searching and manual screening are reported; exact final search date is not stated.
Search strategy	7	Provide the complete search strategies for all databases and other sources, including filters and limits.	Section 2.2; Search concepts, keywords, Boolean operators and publication limits are reported; full database-specific search strings are not provided.
Selection process	8	Describe the study-selection process, independent screening, disagreement resolution, and any automation tools.	Section 2.3, Study Selection; Figure 1. Two independent reviewers screened records. Disagreements were resolved by consensus.
Data collection process	9	Describe the data-collection process, independent extraction/verification, author contact, and automation if used.	Section 2.3, Study Selection; Figure 1. Two independent reviewers screened records. Disagreements were resolved by consensus.
Data items	10a	Define the outcomes or performance measures for	Sections 2.4-2.5; Outcomes/metrics include

		which data were sought and explain how multiple eligible results were handled.	accuracy, precision, recall, F1-score, Dice, IoU, mIoU, AUC and related indices.
	10b	Define other variables extracted and state any assumptions made about missing or unclear information.	Section 2.5; Publication details, crop, disease, region, dataset, sensing modality, model, fusion strategy, metrics, findings, strengths and limitations were extracted
Study risk of bias assessment	11	Describe the method used to assess risk of bias or methodological quality, including reviewers and independence.	Section 2.6, Quality Assessment. Quality domains are described; no named risk-of-bias tool or study-level assessment table is reported.
Effect measures	12	Specify the effect measure(s) used for each outcome in any synthesis.	Sections 2.4-2.5; Study-reported performance metrics were compared descriptively; no pooled effect measure was calculated.
Synthesis methods	13a	Explain how studies were judged eligible for each synthesis or comparison.	Sections 2.4-2.5; Eligible studies were grouped for qualitative and descriptive comparative synthesis according to review scope and extracted characteristics.
	13b	Describe data preparation for synthesis, including handling of missing data and conversions.	Section 2.5; No data conversion, imputation, or transformation procedure is explicitly reported.
	13c	Describe methods used to tabulate or visually present individual-study and synthesis results.	Section 2.5; results are presented in Figures 1-5 and Tables 2,3,5 and 6 across the Results/Discussion sections.
	13d	Describe the synthesis method(s) and explain why the chosen approach was appropriate.	Section 2.5; Qualitative synthesis and descriptive comparative analysis were used because of substantial heterogeneity; no formal meta-analysis was performed.
	13e	Describe methods used to explore possible causes of heterogeneity.	N/A - no subgroup analysis or meta-regression was performed. Heterogeneity is discussed qualitatively in Section 2.5 and Section 5.4
	13f	Describe any sensitivity analyses performed to assess the robustness of synthesized results.	N/A - no statistical sensitivity analysis was conducted because no formal meta-analysis was performed.
Reporting bias assessment	14	Describe methods used to assess bias due to missing	No formal reporting-bias/publication-bias assessment

		results or selective reporting.	method is reported in the current manuscript.
Certainty	15	Describe methods used to assess certainty or confidence in the body of evidence.	No formal certainty-of-evidence assessment framework is reported in the current manuscript.
**RESULT**			
Study selection	16a	Report the results of the search and selection process from identified records to included studies, ideally with a flow diagram.	Figure 1; The identification, screening, eligibility and inclusion stages are shown; numerical record/study counts are not displayed.
	16b	Cite studies that appeared eligible but were excluded after full-text review and explain the reasons for exclusion.	Figure 1;lists general exclusion categories; individual potentially eligible excluded studies and reasons are not cited separately.
Study characteristics	17	Cite every included study and present its main characteristics.	Representative study characteristics are presented in Sections 3-7 and Tables 2, 3 and 5; a complete study-by-study characteristics table is not provided.
Risk of bias in studies	18	Present the risk-of-bias or methodological-quality assessment for each included study.	Section 2.6; describes quality-assessment criteria; study-level risk-of-bias/quality results are not presented.
Results of individual studies	19	Present relevant results for each included study, including outcome information appropriate to the synthesis.	Sections 3-7; and Tables 2, 3 and 5 present selected study findings and reported performance metrics; no pooled effect estimates or precision measures were calculated.
Results of syntheses	20a	Summarize the characteristics and risk-of-bias/quality of studies contributing to each synthesis.	Sections 5.4 and 8, summarise study heterogeneity, methodological limitations and evidence gaps; risk of bias is not summarised for each synthesis.
	20b	Report results of each statistical synthesis, including summary estimates and precision.	N/A - no formal statistical meta-analysis or pooled statistical synthesis was conducted (Section 2.5).
	20c	Report results of investigations of possible causes of statistical heterogeneity.	N/A - no formal statistical investigation of causes of heterogeneity was conducted; heterogeneity is discussed qualitatively.
	20d	Report results of sensitivity analyses.	N/A - no statistical sensitivity analyses were conducted.

Reporting biases	21	Present assessments of risk of bias due to missing results.	No formal assessment of reporting bias due to missing results is presented.
Certainty of evidence	22	Present assessments of certainty or confidence in the evidence for each outcome.	No formal certainty-of-evidence assessment is presented.
**DISCUSSION**			
Discussion	23a	Interpret the results in the context of other evidence.	Sections 5-8; and Section 9, Conclusion.
	23b	Discuss limitations of the evidence included in the review.	Section 5.4; and Section 8; discuss heterogeneity, dataset bias, field generalisation, validation and deployment limitations.
	23c	Discuss limitations of the review process itself.	A dedicated discussion of limitations of the review process itself is not explicitly reported in the current manuscript.
	23d	Discuss implications of the results for practice, policy, and future research.	Section 8, Key Challenges, Research Gaps, and Future Directions; Section 9
**OTHER INFORMATION**			
Registration and protocol	24a	Provide registration information and registration number, or state that the review was not registered.	Not reported in the current manuscript. No review registration statement or registration number is provided.
	24b	Indicate where the review protocol can be accessed, or state that a protocol was not prepared.	Not reported in the current manuscript. No protocol-access statement or statement that a protocol was not prepared is provided.
	24c	Describe and explain amendments to the registered protocol or review plan.	N/A / not reported. Applicability depends on whether a protocol or registration existed.
Support	25	Describe financial or non-financial support and the role of funders or sponsors.	Funding statement; Funding sources are listed; the role of the funders/sponsors in the review is not described.
Competing interests	26	Declare competing interests of the review authors.	Conflicts of Interest, the authors declare no conflicts of interest.
Availability of data, code and other materials	27	State which review materials, data, extraction forms, analytic code, or other materials are publicly available and where.	Section 2.1; identifies Supplementary Table S1 (PRISMA 2020 checklist).
